# Intracellular domain of CATSPER1 could serve as a cytoplasmic platform for redox processes in mammalian sperm

**DOI:** 10.5713/ab.24.0631

**Published:** 2024-12-13

**Authors:** Jingon Kim, Jae Yeon Hwang

**Affiliations:** 1 Department of Integrated Biological Science, Pusan National University, Busan 46241, Korea; 2 Department of Molecular Biology, Pusan National University, Busan 46241, Korea; 3 Institute of Systems Biology, Pusan National University, Busan 46241, Korea

**Keywords:** Antioxidation, CatSper, Interactome, Redox, Sperm

## Abstract

**Objective:**

Mammalian sperm acquire fertilizing ability in the female reproductive tract and develop hyperactivated motility, which is indispensable for male fertility. Hyperactivated motility is initiated by Ca^2+^ influx via the sperm-specific ion channel, CatSper. CATSPER1, a CatSper pore subunit, possesses a long N-terminal intracellular domain and its degradation correlates with unsuccessful sperm migration in the female tract. However, the cellular function and molecular significance of the CATSPER1 N-terminal domain are not well understood. Here, we identify the interactome of the CATSPER1 N-terminal domain and propose a function for the intracellular domain in mammalian sperm.

**Methods:**

To identify CATSPER1 N-terminus interactome, we produced recombinant CATSPER1-N-terminus in bacterial system. The purified protein was incubated with testicular lysates and eluted together with testicular interacting proteins. The elutes were subjected to proteomic analysis and CATSPER1-N-terminus interactome was profiled. Identified proteins were further analyzed by functional annotation.

**Results:**

We purified the partial CATSPER1 N-terminal domain and identified 57 testicular proteins as domain interactomes using mass spectrometry analysis. Functional annotation analysis revealed that 106 gene ontologies were significantly enriched, 16 of which were related to redox processes. We found that antioxidant enzymes, such as PARK7 and PRDX2, 4, and 6, were included in the enriched redox-related gene ontologies.

**Conclusion:**

These results suggest that the CATSPER1 N-terminus could function in defending against oxidative stress to support the successful migration of mammalian sperm to fertilizing sites in the female reproductive tract.

## INTRODUCTION

Ejaculated mammalian sperm gradually acquire fertilizing ability in the female reproductive tract through a process termed capacitation [1] Capacitated mammalian sperm develop a unique motility pattern called hyperactivated motility, which is characterized by asymmetric flagellar beating with an increased angle of flagellar curvature [2]. Mammalian sperm can reach the ampulla, the fertilizing site, by developing hyperactivated motility, and penetrate the zona pellucida, a glycoprotein barrier of oocytes, to achieve successful fertilization [3,4]. This unique sperm motility pattern, which is crucial for male fertility, is initiated by Ca^2+^ influx [5] via the sperm-specific ion channel, called CatSper [3,6]. Previous studies have demonstrated that genetic alterations of the CatSper channel prevent sperm from developing hyperactivated motility, which compromises male fertility in both mice and humans [7]. In addition, reduced intracellular Ca^2+^ levels due to limited activation of the CatSper channel have been observed in infertile male patients [8]. These studies have elucidated the physiological significance of CatSper channels in male fertility.

In humans and mice, intracellular alkalinization is important for activating CatSper channels to introduce extracellular Ca^2+^ into the sperm [9,10]. A previous study demonstrated that non-transmembrane (TM) CatSper subunits, EFCAB9 and CATSPERζ, form a binary complex which senses increasing intracellular Ca^2+^ in a pH-dependent manner to further activate the CatSper channel [11,12]. Thus, genetic depletion of either EFCAB9 or CATSPERζ alters sperm hyperactivation, impairing male fertility [11,12]. These studies demonstrate that fine-tuned molecular regulation of Ca^2+^ influx, as the first step, is critical for the development of hyperactivated motility in mammalian sperms. However, how the initiated signaling by the introduced Ca^2+^ via the CatSper channel is molecularly transduced from the peri-axonemal space to the axoneme is not well understood.

The CatSper channel is a multiprotein complex composed of at least 14 subunits [13–16]. CATSPER1, 2, 3, and 4 are six TM proteins that form a heterotetrameric pore of the CatSper channel complex. Interestingly, pore subunits have larger intracellular domains than the other CatSper TM subunits [6]. Previous studies have demonstrated that the intracellular domain of CATSPER1 is subjected to proteolysis by Ca^2+^ influx, which is observed in the majority of capacitated sperm, except for successfully hyperactivated sperm [17,18]. These results highlight that the intracellular domain of CATSPER1 could contribute to changes in downstream signaling and/or sperm physiology after Ca^2+^ influx in capacitating sperm. However, the detailed roles of the CATSPER1 N-terminal domain have not yet been elucidated.

In here, we purified the recombinant CATSPER1 N-terminal domain and identified its interacting proteins in the male germline, suggesting its potential contribution to the molecular and physiological changes in capacitating sperm by CatSper-mediated Ca^2+^ signaling. We identified the testicular interactome of the CATSPER1 N-terminal domain and performed functional annotation to determine its potential role in CatSper-associated signaling and physiology in mammalian sperm. This study explores the molecular association of the CATSPER1 intracellular domain and suggests a molecular function of CATSPER1 proteolysis, which correlates with sperm migration in the female reproductive tract [17].

## MATERIALS AND METHODS

### Animals

Wild-type C57BL/6 mice were purchased from Samtako Bio-Korea, Inc. (Osan, Korea ). The mice were managed according to the approved guidelines of the Institute of Animal Care and Use Committee of Pusan National University (#PNU-2023-0386).

### Recombinant protein expression

The BL21(DE3)-Rossetta bacterial strain (Enzynomics, Daejeon, Korea) was used to express recombinant proteins. The *pGEX-6P2* and *pGEX-2T-Catsper1-N150* constructs, which encode a glutathione S-transferase (GST) tag and mouse CATSPER1 fragments (1 to 150 amino acids) tagged with GST at the N-terminus, were transformed into BL21(DE3)-Rossetta competent cells. Freshly formed colonies were picked and inoculated into Luria broth (KisanBio, Seoul, Korea) supplemented with 50 μg/mL ampicillin (Sigma-Aldrich, St. Louis, MO, USA) and 12.5 μg/mL chloramphenicol (KisanBio), followed by overnight culture at 37°C. Saturated cultivates were inoculated into terrific broth supplemented with 50 μg/mL ampicillin at a 1:50 ratio (v/v) and were further cultured at 37°C. Next, 0.5 mM of isopropyl-1-thio-β-D-galactopyranoside ([IPTG] KisanBio) was added when the optical density (OD)_600_ absorbance values of the cultivates reached 0.5 to 0.8, to induce recombinant protein expression. After IPTG addition, the bacterial cells were cultured at 15°C for 15 to 17 hours.

### Recombinant protein purification

Bacterial protein purification was performed as previously described [12,19]. Briefly, IPTG-induced bacterial cells were harvested and washed with 1X phosphate-buffered saline (10 mM phosphate, 140 mM NaCl, pH 7.5). The washed cells were resuspended in 2X tris-buffered saline (TBS, 40 mM Tris, 300 mM NaCl, pH 7.5) supplemented with 0.5 X protease inhibitor cocktail (MedChemExpress, Monmouth Junction, NJ, USA) and lysed by sonication. Lysates were centrifuged at 4°C and 18,000×g for 1 hour to separate the soluble and insoluble pellet fractions. Supernatants were collected and incubated with Pierce glutathione agarose (ThermoFisher Scientific, Waltham, MA, USA) at 4°C overnight with gentle rocking. The agarose resin was washed time with 2X TBS supplemented with 1% Triton X-100 (Sigma-Aldrich) and eluted with 1X TBS supplemented with 10 mM reduced glutathione (Enzo Life Science, Farmingdale, NJ, USA), pH 7.5. Elutes were dialyzed against 1X TBS in 50% glycerol solution, pH 7.5 at 4°C, and stored at −20°C until use.

### Gel staining and immunoblotting

Bacterial proteins from the lysates and purified recombinant proteins were subjected to gel staining and immunostaining. For the solubility test, separated soluble and insoluble fractions of bacterial lysates were mixed with 2X lithium dodecyl sulfate (LDS) equivalently and denatured by heating at 75°C for 10 minutes supplemented with 50 mM dithiothreitol ([DTT] ThermoFisher Scientific). Purified recombinant proteins were denatured using 1X LDS supplemented with 50 mM DTT and heated at 75°C for 2 minutes. Denatured proteins were subjected to sodium dodecyl sulfate-polyacrylamide gel electrophoresis (SDS-PAGE), followed by either gel staining or immunoblotting. Coomassie blue gel staining and silver staining were performed using Imperial Protein Stain (ThermoFisher Scientific) and Pierce Silver Stain Kit (ThermoFisher Scientific), respectively, according to the manufacturer’s instructions. Immunoblotting was performed as previously described [19,20]. Briefly, proteins in SDS-PAGE gels were transferred to a 0.45 μM nitrocellulose membrane (BioRad, Hercules, CA, USA) followed by blocking the membrane using 5% skim milk (Becton, Dickinson and Company, Franklin Lakes, NJ, USA) in 1X TBS supplemented with 0.1% Tween-20 ([TBS-T] Duksan, Ansan, Korea) for an hour at room temperature (RT). The blocked membranes were incubated with mouse monoclonal anti-GST (SC-138; 1:2,000) antibody from SantaCruz Biotechnology (Dallas, TX, USA) at 4°C overnight. The membranes were washed three times with TBS-T for 10 minutes each, followed by incubation with goat anti-mouse IgG conjugated with horseradish peroxidase (115-035-003; 1:20,000) from Jackson ImmunoResearch (West Grove, PA, USA). The membranes were washed again with TBS-T three times at RT for 10 minutes each and developed using SuperSignal West Dura solution (ThermoFisher Scientific).

### Testis interactome identification

#### Interactome sample precipitation

Testicular protein solubilization was performed as previously described [12,20]. Briefly, testes were homogenized in 0.32M sucrose using a dounce homogenizer and centrifuged at 4°C, 1,500×g for 10 minutes. The supernatant was collected and lysed with 0.5% Triton X-100 supplemented with 1X protease inhibitor (MedChemExpress, Monmouth Junction, NJ, Korea) with brief sonication, followed by gentle rocking at 4°C for 3 to 4 hours. The lysates were centrifuged at 18,000×g at 4°C for 1 hour and the supernatant was collected. The collected supernatant was 10 times diluted with 1X TBS and filtered. The solubilized testicular proteins were incubated with immobilized recombinant GST or GST-tagged CATSPER1-N150 proteins, which were prepared by incubation with glutathione agarose (Pierce glutathione agarose, ThermoFisher Scientific) at 4°C overnight. The incubated mixtures were washed with 1X TBS and eluted with 1X TBS supplemented with 10 mM reduced glutathione, pH7.5. The collected elutes were precipitated using trichloroacetic acid (Sigma-Aldrich). The precipitates were dissolved in 8 M urea and denatured in 1X LDS buffer supplemented with 50 mM DTT, followed by heating at 75°C for 5 minutes. Denatured proteins were subjected to SDS-PAGE, followed by Coomassie-blue gel staining for liquid chromatography-tandem mass spectrometry (LC-MS/MS).

#### Liquid chromatography-tandem mass spectrometry and interacting testicular protein identification

Coomassie-stained gel pieces were placed in a destain buffer containing 50% CH_3_CN and 25 mM NH_4_HCO_3_ and centrifuged twice at 37°C and 750 rpm for 10 minutes. Destained gels were dehydrated with 100% NH_4_HCO_3_ and incubated with 50 mM NH_4_HCO_3_ buffer containing 10 mM DTT at 56°C for 30 min for reduction. After reduction, proteins in the gels were incubated with alkylated by incubation with 50 mM NH_4_HCO_3_ buffer containing 25 mM iodoacetamide at RT for 30 minutes followed by dehydration with 100% CH_3_CN three times. Proteins in the gels were trypsinized for 16 hours at 37°C, and the peptides were digested and purified with a SOLA HRP 96 well plate C18 cartridge (ThermoFisher Scientific). Purified peptides were reconstituted in 0.1% trifluoroacetic acid and subjected to mass spectrometry analysis using Orbitrap Exploris 480 coupled with Ultimate 3000 ultra-high performance liquid chromatography (ThermoFisher Scientific). The detected peptides were analyzed using data-dependent acquisition methods. The resolutions were set to 60,000 and 30,000 for the first and second rounds of mass spectrometry analysis, respectively. The collected data were processed using the SAGE open-search engine and mouse protein database (UniProt ID: UP000000589). Peptides with lengths from 7 to 50 residues were only subjected to queries, and a 1% false discovery rate was applied to the spectrum, peptide, and protein levels in this study. Proteins with spectral values greater than two-fold in GST-CATSPER1-N150 compared to the values in recombinant GST were considered to interact with CATSPER1-N150.

### Functional annotation

Identified CATSPER1-N150-interactomes were subjected to functional annotation using the Database for Annotation, Visualization, and Integrated Discovery (DAVID; https://david.ncifcrf.gov/). Gene ontologies with fold enrichments greater than two-fold and p-values lower than 0.05 were considered significant. The enriched ontologies were categorized using REVIGO (http://revigo.irb.hr) and visualized using R software. Tissue expression profiles of CATSPER1-N150-interacting proteins included in the enriched ontologies were obtained from the ENCODE project in the National Center for Biotechnology Information gene (NCBI) Gene database (https://www.ncbi.nlm.nih.gov/gene).

## RESULTS

### Recombinant mouse CATSPER1 N-terminal domain is purified

**Figure 1:**
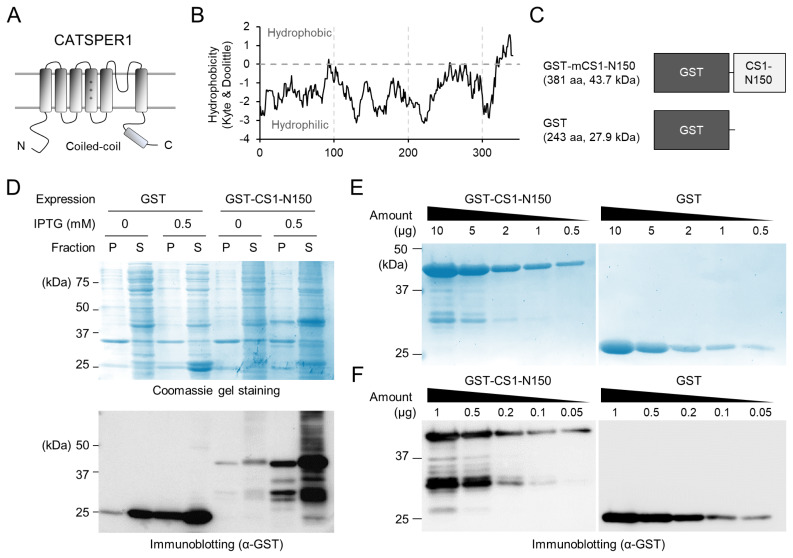
The partial N-terminus intracellular domain of mouse CATSPER1 is purified. (A) Topology of mouse CATSPER1. A cartoon depicts the mouse CATSPER1 topology composed of six transmembrane (TM) domains and N- and C-terminal intracellular domains. Voltage-sensing TM is marked with “+”. (B) Hydrophobicity analysis of the N-terminus intracellular domain for mouse CATSPER1. Shown is a hydropathy plot for the N-terminal intracellular domain (351 amino acids) of mouse CATSPER1 (Uniprot: Q91ZR5) calculated using the Kyte and Doolittle hydrophobicity scale is shown. Window size = 9 amino acids. (C) Diagrams for recombinant mCATSPER1 (CS1) N-terminus (1 to 150 aa) tagged with GST (GST-mCATSPER1-N150) and free GST used in this study. (D) Solubility of the recombinant CATSPER1 N-terminal domain (GST-CS1-N150) and GST in the bacterial system. Partitioning of the recombinant proteins in the pellet (P) or soluble (S) fractions is examined by Coomassie gel staining (top) and immunoblotting (bottom). (E–F) Purification of the recombinant CATSPER1 N-terminus intracellular domain (GST-CS1-N150, left) and free GST (right). Purified recombinant proteins were confirmed by Coomassie gel staining (E) and immunoblotting (F). α-GST was used for immunoblotting to detect the recombinant proteins (D and F). GST, glutathione S-transferase; IPTG, isopropyl-1-thio-β-D-galactopyranoside.

The N-terminus intracellular domain of mouse CATSPER1 undergoes truncation and proteolytic degradation during capacitation *in vitro* and in the female tract [17,18]. To identify the proteins interacting with the N-terminus of CATSPER1, we purified the CATSPER1 N-terminus (Figure 1). CATSPER1 carries six TM domains, and its N-terminal intracellular domain is 351 amino acids long (UniProt: Q91ZR5; Figure 1A). Because the full-length CATSPER1 N-terminus was not solubilized in the bacterial system, we purified an intracellular domain fragment of 150 amino acids (1 to 150 amino acids) with high hydrophilicity (Figures 1B, 1C). As in previous studies, the GST-tagged mouse CATSPER1 N-terminal domain (GST-mCATSPER1-N150) expresses well and is highly soluble in the bacterial system (Figure 1D) [3,12]. Coomassie blue gel staining demonstrated that the recombinant GST-mCATSPER1-N150 was successfully purified (Figure 1E). Immunoblotting analysis revealed that the recombinant GST-mCATSPER1-N150 was partly truncated and purified together (Figure 1F). Recombinant free GST protein was also purified and used as a negative control.

### Testicular proteins to interact with the mouse CATSPER1 N-terminus domain are identified

**Figure 2:**
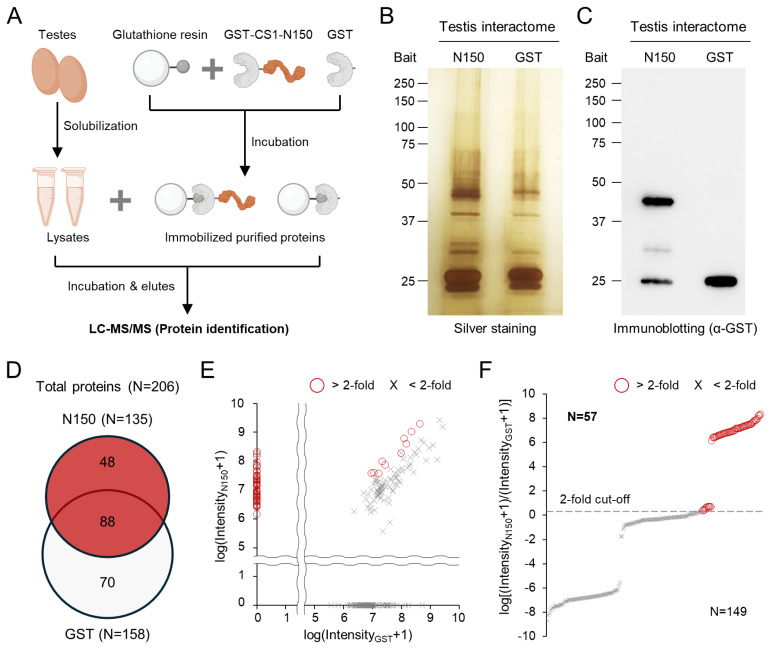
Testicular interactome of the mouse CATSPER1 N-terminal domain is identified. (A) A schematic diagram depicting the identification of testicular proteins interacting with recombinant GST-mCATSPER1-N150 (GST-CS1-N150) and free GST. (B and C) Silver staining (B) and immunoblotting (C) of the testicular interactome of recombinant GST and GST-mCATSPER1-N150. (D–F) Quantitative analyses of testicular proteins interacting with the mouse CATSPER1 N-terminus domain. (D) A Venn-diagram represents the number of testicular proteins interacting with recombinant GST (light gray, N = 158) and GST-mCATSPER1-N150 (red, N = 135) identified from mass spectrometry analyses. (E and F) Identified proteins are mapped in accordance with their spectra intensity in GST and GST-mCATSPER1-N150 (E; x- and y-axis, respectively) or relative ratio (F; y-axis). Proteins of which fold changes in GST-mCATSPER1-N150 over GST are above 2 were considered to interact with mCATSPER1-N150 specifically (red circles, N = 57; E and F). GST, glutathione S-transferase; LC-MS/MS, liquid chromatography-tandem mass spectrometry.

Sperm are highly differentiated cells with a marginal amount of cytosol, which limits their protein solubilization. Thus, we used adult testes, which possess male germ cells at different stages of development, including testicular sperm, to obtain sufficient solubilized germline proteins for interactome analysis. To identify the testicular proteins interacting with purified GST-mCATSPER1-N150 (Figure 1), solubilized testis lysates were incubated with the immobilized recombinant proteins and eluted (Figure 2A). Elutes from the free GST incubates were used as negative controls. Silver staining and immunoblotting demonstrated that the elutes contained sufficient interacting proteins for LC-MS/MS analysis (Figures 2B, 2C). Each elute was subjected to LC-MS/MS analysis to identify testicular proteins, with profiling intensity values for individual proteins (Figures 2D–2F; Supplementary Table S1). A total of 206 proteins were detected from both elutes, and 48 and 71 proteins were detected to interact with GST-mCATSPER1-N150 and free GST, respectively (Figure 2D). Among the 88 proteins detected in both elutes, the intensity values of nine proteins were twice higher in elutes from GST-mCATSPER1-N150 than in those from free GST (Figures 2E, 2F; Supplementary Table S1). The 57 testicular proteins were considered to testicular interactome of the mouse CATSPER1 N-terminus domain in this study (Figure 2F). In particular, the identified interactome includes a couple of proteins to modulate glutathione metabolism (GLRX, GSTA2, GSTM3, GSTM4, GSTM6, and GSTM7), antioxidation (PRDX2, PRDX4, PRDX5, PARK7, and LANCL1), and reducing process (AKR7A2, AKR1CL, AKR1B1, FKBP4, and PDIA6) (Table 1). Also, several proteins which participate in carbohydrate metabolism (GAPDH, GAPDHS, FBP1, LDHC, and PGP) were also identified as CATSPER1-N150 interactome from the analyses (Table 2).

### N-terminus domain of the CATSPER1 is involved in redox events in sperm

**Figure 3:**
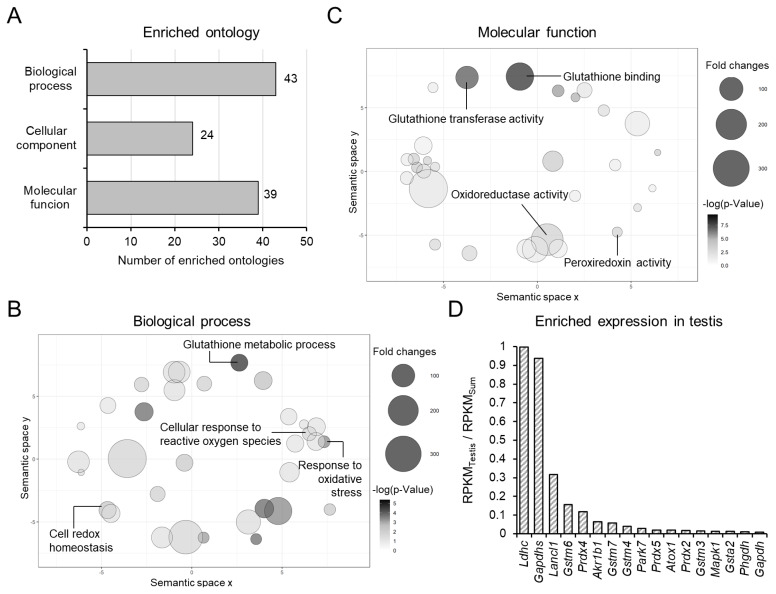
Testicular interactome of the mouse CATSPER1-N150 is significantly enriched in gene ontologies related to redox processes. (A–C) Enriched gene ontologies of the mouse CATSPER1-N150 interactome in the testis. (A) Numbers of gene ontologies in three categories: biological process (N = 43), cellular component (N = 24), and molecular function (N = 39) are shown in the bar plot. (B–C) Biological process (C) and molecular function (C) gene ontologies were functionally categorized. Significantly enriched ontologies are marked as bubbles in the scatter plot. The colors and sizes of the bubbles indicate the p-values and fold changes, respectively, in each ontology. (D) Testicular enrichment of mRNAs encoding the mCATSPER1-N150 interactome involved in redox processes. Enriched testicular expression of the targets was calculated by normalizing the RPKM values in the testis (RPKM_Testis_) with the sum of the RPKM values from adult tissues (RPKM_Sum_). Mouse tissue expression data were obtained from the NCBI Gene database. NCBI, National Center for Biotechnology Information gene; RPKM, reads per kilobase of per million mapped reads.

To understand the potential function of the N-terminal intracellular domain of CATSPER1, we performed functional annotation of the identified mCATSPER1-N150 interactome (Figure 3; Supplementary Table S2). In total, 106 gene ontologies were significantly enriched in the functional annotation study (Figure 3A). Interestingly, each eight gene ontologies in the biological process and molecular function categories were related to redox processes, such as glutathione metabolism and cellular response to oxidative stress (Figures 3B, 3C; Table 3). A total of 17 proteins were included in the enriched 16 gene ontologies related to the redox process. Especially, several peroxiredoxins (PRDX2, 4, and 5) and μ types of glutathione S-transferases (GSTM3, 4, 6, and 7) were also identified which are included in the significantly enriched ontologies. Among the 17 proteins, genes encoding LDHC, GAPDHS, LANCL1, GSTM6, and PRDX4 were dominantly expressed in the testis, which occupied over 10% of the total expression level in all tissues of adult mice (Figure 3D). These results elucidate that the N-terminus of CATSPER1 could be significantly involved in redox-related biological and molecular processes in mammalian sperm.

## DISCUSSION

After ejaculation, over tens of millions of mammalian sperm initiate to travel to fertilizing sites in the female reproductive tract. In the female reproductive tract, sperm undergo a variety of biochemical and physiological changes and acquire fertilizing ability via capacitation process [1]. During capacitation, mammalian sperm develop hyperactivated motility [2], which enables sperm to pass through the utero-tubal junction (UTJ) and penetrate the zona pellucida of the oocytes for successful fertilization [3,17,18]. However, only a small portion of ejaculated sperm can successfully develop hyperactivated motility and meet eggs in a timely manner.

Ca^2+^ influx via CatSper is the key trigger for sperm hyperactivation in mammalian sperm [3,6,14,21]. Previous studies have demonstrated that genetic ablation of the CatSper channel results in unsuccessful sperm hyperactivation and failure to pass through the UTJ in the female tract [6]. However, even in fertile males, only a limited number of ejaculated sperm reach the fertilizing site, indicating robust selection mechanisms induced by the female tract environment and/or the sperm themselves. Interestingly, previous studies observed that CATSPER1 undergoes proteolytic degradation during capacitation [17,18]. In particular, only capacitated sperm with an intact distribution of preserved CATSPER1 along the sperm tail can successfully develop hyperactivated motility *in vitro* [18]. A recent study demonstrated that the N-terminal intracellular domain of CATSPER1 is a target of proteolytic events triggered by Ca^2+^ influx [17]. Furthermore, *in situ* imaging of the reproductive tract of mated female mice revealed that almost all sperm that reach the fertilizing site maintain the intact CATSPER1 N-terminus; however, others lack the intracellular domain of CATSPER1 [17]. These results indicate that the N-terminal domain of CATSPER1 is highly correlated with sperm physiology and downstream signaling pathways initiated by Ca^2+^ influx in the female tract.

In our study, the N-terminal intracellular domain of CATSPER1 interacted with multiple proteins in the testis (Figure 2). Considering the correlation between CATSPER1 proteolysis and unsuccessful sperm migration, the identified interactome could be associated with Ca^2+^ signaling in sperm and/or sperm physiology. In particular, over 10% of the enriched gene ontologies are related to redox systems, such as antioxidation processes, which are crucial for sperm function in various mammals [22,23]. Previous studies have demonstrated that suppressing reactive oxygen species (ROS) by antioxidant treatment can improve sperm motility and fertility *in vitro* in mammals, including domestic animals [22]. Although sperm cells are quite vulnerable to oxidative stress, they actively generate ROS during capacitation because of their limited antioxidant enzymes [23]. Those ROSs are primarily generated by metabolic processes in the mitochondria, which are further activated during capacitation [22,23]. Thus, capacitating sperm usually possess high levels of ROS, which eventually causes sperm apoptosis [23]. Considering that sperm with an altered CATSPER1 N-terminus, which interacts with antioxidation enzymes (Figure 3; Table 1), such as PARK7 [24], PRDX2 [25], PRDX4 [26], and PRDX6 [27], fail to arrive at a fertilizing site [17], the CATSPER1 N-terminus could serve as a platform to protect against oxidative stress during sperm capacitation.

Impaired Ca^2+^ influx results in robust global tyrosine phosphorylation (pTyr) during sperm capacitation [18]. This post-translational modification of sperm proteins is activated by soluble adenylyl cyclase (sAC), followed by PKA signaling cascades [28]. Interestingly, ROSs are known to enhance sAC activity to generate more cAMPs and suppress tyrosine phosphatase activity [29]. Thus, ROSs could synergistically elevate pTyr levels in capacitated sperm. A previous study observed that a smaller number of sperm in the ampulla potentiated pTyr compared to sperm in the UTJ [17]. Simultaneously, the CATSPER1 N-terminus, which interacts with antioxidant enzymes (Figure 3), is degenerated in the majority of sperm that fail to arrive at the ampulla. Thus, sperm that fail to reach the fertilization site undergo CATSPER1 N-terminal proteolysis, presumably due to premature Ca^2+^ entry [17,30], and proteolysis could alter antioxidant enzyme function, followed by ROS elevation and increased pTyr potentiation. This suggests that proteolysis of the CATSPER1 N-terminal domain could be involved in endogenous selection mechanisms by regulating oxidative stress via its interaction with antioxidant enzymes in ejaculated sperm. The detailed regulatory mechanisms to protect against oxidative stress by CATSPER1-mediated antioxidation enzymes require further explored.

In domestic animals, artificial insemination is widely employed to produce livestock [31]. Despite its successful establishment in the fields, there have been continued efforts to enhance success rates by improving sperm quality and fertility [31,32]. Especially, reducing oxidative stress by treating antioxidants significantly enhances sperm motility and fertility in domestic animals [22]. Our study suggests that CATSPER1 N-terminus and its degradation by premature Ca^2+^ influx [17] could be associated with antioxidation process in capacitating sperm. Previous studies identified CATSPER subunits in sperm from domestic animals [14]. In addition, pharmacological approaches demonstrated that CatSper inhibition significantly impairs hyperactivated motility in bull [33], pig [34], and horse [35] sperm, elucidating physiological importance of CatSper channel in domestic animals. Despite the limited understanding for molecular characteristics of CatSper channel, its conserved physiological significance suggests that CATSPER1 N-terminus could contribute to modulating oxidative stresses in capacitating sperm from domestic animals. Therefore, understanding molecular association of the CATSPER1 N-terminus and antioxidation in capacitating sperm could provide new strategies to manage oxidative stresses in sperm with improving their quality, which is expected to advance artificial insemination technology in domestic animals.

## CONCLUSION

In this study, we identify the interactome for CATSPER1 N-terminus, which undergoes proteolytic cleavage and degradation in sperm failed to reach fertilizing sites. Of note, the target region for proteolysis interacts with several antioxidation enzymes and redox-associated proteins to regulate oxidative stresses, which should be suppressed in capacitating sperm for successful migration and fertilization. Thus, we conclude that CATSPER1 N-terminus could function in platform for antioxidation enzymes to avoid oxidative stress in capacitating sperm. Also, we conclude that the proteolytic degradation of CATSPER1 N-terminus is an endogenous sperm selection mechanism, which could be determined by apoptosis-inducing oxidative stress triggered by abnormal Ca^2+^ homeostasis in capacitating sperm. This study provides new insights for the association between oxidative stress and Ca^2+^ signaling with expanding mechanistic significance of the CatSper-mediated Ca^2+^ signaling in function and selection of mammalian sperm.

## Figures and Tables

**Table 1:** Redox-related proteins interacting with CATSPER1-N150 in testis

Categories	Protein	Uniprot ID	Description	Log (GST+1)	Log (N150+1)	Log ([N150+1]/[GST+1])
Glutathione metabolism	GSTM3	spP19639	Glutathione S-transferase Mu 3	0.000	7.502	7.502
	GLRX	spQ9QUH0	Glutaredoxin-1	0.000	7.156	7.156
	GSTA2	spP10648	Glutathione S-transferase A2	0.000	6.585	6.585
	GSTM6	spO35660	Glutathione S-transferase Mu 6	8.346	9.026	0.680
	GSTM7	spQ80W21	Glutathione S-transferase Mu 7	8.627	9.306	0.679
	GSTM4	spQ8R5I6	Glutathione S-transferase Mu 4	8.175	8.615	0.440
Antioxidation	PRDX2	spQ61171	Peroxiredoxin-2	0.000	7.514	7.514
	PARK7	spQ99LX0	Parkinson disease protein 7 homolog	0.000	7.492	7.492
	PRDX4	trB1AZS9	Peroxiredoxin 4	0.000	7.261	7.261
	PRDX5	spP99029	Peroxiredoxin-5, mitochondrial	0.000	6.609	6.609
	LANCL1	spO89112	Glutathione S-transferase LANCL1	8.097	8.788	0.690
Reducing process	AKR7A2	spQ8CG76	Aflatoxin B1 aldehyde reductase member 2	0.000	7.190	7.190
	AKR1CL	trG3XA14	Aldo-keto reductase family 1, member C-like	0.000	7.174	7.174
	AKR1B1	spP45376	Aldo-keto reductase family 1 member B1	0.000	6.757	6.757
	FKBP4	spP30416	Peptidyl-prolyl cis-trans isomerase FKBP4	0.000	6.491	6.491
	PDIA6	spQ922R8	Protein disulfide-isomerase A6	0.000	6.463	6.463

**Table 2:** Metabolism-regulating testicular proteins interacting with CATSPER1-N150 in testis

Protein	Uniprot ID	Description	Log (GST+1)	Log (N150+1)	Log ([N150+1]/[GST+1])
PGP	spQ8CHP8	Glycerol-3-phosphate phosphatase	0.000	7.476	7.476
GAPDHS	spQ64467	Glyceraldehyde-3-phosphate dehydrogenase, testis-specific	0.000	6.961	6.961
GAPDH	spP16858	Glyceraldehyde-3-phosphate dehydrogenase	7.334	8.004	0.670
FBP1	spQ9QXD6	Fructose-1,6-bisphosphatase 1	6.958	7.593	0.635
LDHC	spP00342	L-lactate dehydrogenase C chain	7.215	7.573	0.359

GST, glutathione S-transferase.

**Table 3:** Enriched gene ontologies for CATSPER1-N150 interactome associated with redox processes

Categories	Term	Fold enrichment	p-value	Proteins
Biological process	GO:0006749-glutathione metabolic process	45.980	4.059E-06	GSTM4, GSTM3, GSTA2, GSTM7, GSTM6
	GO:0006979-response to oxidative stress	18.075	1.607E-04	PRDX2, PRDX5, PRDX4, PARK7, ATOX1
	GO:0042744-hydrogen peroxide catabolic process	52.418	1.451E-03	PRDX2, PRDX5, PRDX4
	GO:0045454-cell redox homeostasis	47.652	1.755E-03	PRDX2, PRDX5, PRDX4
	GO:0034614-cellular response to reactive oxygen species	26.653	5.513E-03	PRDX5, MAPK1, PARK7
	GO:0034599-cellular response to oxidative stress	14.976	1.665E-02	PRDX2, PRDX5, PARK7
	GO:0042743-hydrogen peroxide metabolic process	116.483	1.674E-02	PRDX2, PARK7
	GO:0033554-cellular response to stress	52.418	3.684E-02	PRDX2, PRDX4

Molecular function	GO:0043295-glutathione binding	153.711	3.274E-10	GSTM4, GSTM3, GSTA2, LANCL1, GSTM7, GSTM6
	GO:0004364-glutathione transferase activity	90.418	5.757E-09	GSTM4, GSTM3, GSTA2, LANCL1, GSTM7, GSTM6
	GO:0140824-thioredoxin-dependent peroxiredoxin activity	307.421	3.598E-05	PRDX2, PRDX5, PRDX4
	GO:0008379-thioredoxin peroxidase activity	219.586	7.537E-05	PRDX2, PRDX5, PRDX4
	GO:0051920-peroxiredoxin activity	219.586	7.537E-05	PRDX2, PRDX4, PARK7
	GO:0016491-oxidoreductase activity	6.798	1.491E-04	LDHC, PRDX2, PRDX5, PRDX4, GAPDHS, AKR1B1, PHGDH, GAPDH
	GO:0016209-antioxidant activity	61.484	1.053E-03	PRDX2, PRDX5, PRDX4
	GO:0004601-peroxidase activity	35.747	3.101E-03	PRDX2, PRDX5, PRDX4

## SUPPLEMENTARY MATERIAL

**Supplementary Table S1.** List of GST-mCATSPER1-N150 and GST- interacting proteins in the testis

**Supplementary Table S2.** Functional annotation of the mCATSPER1-N150 interactome in the testis
