# Supplementary material for: Intracellular domain of CATSPER1 could serve as a cytoplasmic platform for redox processes in mammalian sperm
Source: Anim Biosci. 2024 Dec 13;38(4):655–64. doi: 10.5713/ab.24.0631 (PMC11917441; doi:10.5713/ab.24.0631)
Supplement: Supplementary file 1 [file ab-24-0631-Supplementary-Table-1.pdf]

Supplementary Table S1. List of GST-mCATSPER1-N150 and GST-interacting proteins in the testis

Protein ID	Intensity	
	GST (log2)	GST-mCATSPER1-N150 (log2)
sp|Q9ES34|UBE3B_MOUSE	0	27.73344408
sp|Q62426|CYTB_MOUSE	0	27.44902125
sp|O70251|EF1B_MOUSE	0	27.18700075
sp|Q9R0P5|DEST_MOUSE	0	26.46510585
tr|Q9Z1R9|Q9Z1R9_MOUSE	0	26.13235022
sp|Q8VED5|K2C79_MOUSE	0	26.08556845
sp|O08997|ATOX1_MOUSE	0	25.9278207
sp|P05214|TBA3_MOUSE	0	25.51921482
sp|Q6NXH9|K2C73_MOUSE	0	25.37037215
sp|Q61171|PRDX2_MOUSE	0	24.96198083
sp|P19639|GSTM3_MOUSE	0	24.92016851
sp|Q99LX0|PARK7_MOUSE	0	24.88922056
sp|Q8CHP8|PGP_MOUSE	0	24.83569414
sp|P60335|PCBP1_MOUSE	0	24.65227557
sp|P68372|TBB4B_MOUSE	0	24.52249223
sp|P17751|TPIS_MOUSE	0	24.45285945
sp|Q9CQ43|DUT_MOUSE	0	24.35954757
sp|Q01755|TCP11_MOUSE	0	24.23474323
tr|B1AZS9|B1AZS9_MOUSE	0	24.12149882
sp|P62259|1433E_MOUSE	0	24.02820561
sp|Q9JJI8|RL38_MOUSE	0	23.93928079
sp|Q8CG76|ARK72_MOUSE	0	23.88348322
tr|G3XA14|G3XA14_MOUSE;tr|Q3UXL1|Q3UXL1_MOUSE	0	23.83160533
sp|P68037|UB2L3_MOUSE	0	23.80482586
sp|Q9QUH0|GLRX1_MOUSE	0	23.77216742
sp|Q9Z1Q5|CLIC1_MOUSE	0	23.27551019
sp|P07901|HS90A_MOUSE	0	23.18272331
sp|Q64467|G3PT_MOUSE	0	23.12232814
sp|P11983|TCPA_MOUSE	0	23.04600023
sp|P05213|TBA1B_MOUSE	0	22.90156561
sp|P16460|ASSY_MOUSE	0	22.8339567
sp|P63085|MK01_MOUSE	0	22.72428455
sp|Q8R0P4|AAMDC_MOUSE	0	22.70054533
sp|P80316|TCPE_MOUSE	0	22.59341751
sp|Q99MD9|NASP_MOUSE	0	22.50277921
sp|P45376|ALDR_MOUSE	0	22.44495338
sp|Q9CYG7|TOM34_MOUSE	0	22.38118532
sp|P99024|TBB5_MOUSE	0	22.2646535
sp|P15105|GLNA_MOUSE	0	22.15991253
sp|Q8BKC5|IPO5_MOUSE	0	22.02159072
sp|P99029|PRDX5_MOUSE	0	21.95507999

sp|P10648|GSTA2_MOUSE	0	21.87445591
sp|Q9Z2I9|SUCB1_MOUSE	0	21.64208396
sp|P30416|FKBP4_MOUSE	0	21.56158559
sp|Q922R8|PDIA6_MOUSE	0	21.47106583
sp|P61205|ARF3_MOUSE;sp|P84078|ARF1_MOUSE;sp|Q8BSL7|ARF	0	21.40817924
sp|Q61753|SERA_MOUSE	0	21.33467989
sp|P80317|TCPZ_MOUSE	0	20.50068018
sp|O89112|LANC1_MOUSE	26.89797621	29.19146782
sp|O35660|GSTM6_MOUSE	27.72385771	29.98412845
sp|Q80W21|GSTM7_MOUSE	28.65853136	30.91341792
sp|P16858|G3P_MOUSE	24.36296995	26.59022734
sp|Q9QXD6|F16P1_MOUSE	23.11504644	25.22413902
sp|O08716|FABP9_MOUSE	23.42906469	25.28797074
sp|Q8R5I6|GSTM4_MOUSE	27.15782714	28.61786262
sp|P18760|COF1_MOUSE	24.72477657	26.18248095
sp|P00342|LDHC_MOUSE	23.96615002	25.15716388
sp|Q9JJK2|LANC2_MOUSE	26.54213328	27.54007302
sp|P61079|UB2D3_MOUSE	23.64991368	24.62088894
sp|Q8VC30|TKFC_MOUSE	23.62504829	24.32860349
sp|Q8C1A5|THOP1_MOUSE	22.24628134	22.92852435
sp|P63017|HSP7C_MOUSE	23.69320467	24.37184297
sp|Q60854|SPB6_MOUSE	23.38809478	23.97386567
sp|Q9R0Q7|TEBP_MOUSE	23.73359085	24.09663157
sp|P0CG49|UBB_MOUSE;sp|P0CG50|UBC_MOUSE;sp|P62983|RS27	30.9609529	31.30581729
sp|Q9CQM9|GLRX3_MOUSE	25.36297124	25.65530324
sp|Q9D154|ILEUA_MOUSE	23.097885	23.35963212
sp|P27773|PDIA3_MOUSE	24.05355545	24.26705728
sp|P10649|GSTM1_MOUSE	28.80751357	28.98532221
sp|P48758|CBR1_MOUSE	26.79479542	26.89036718
sp|P09041|PGK2_MOUSE	22.75523907	22.76757346
sp|Q9DCS2|MTL26_MOUSE	24.69231426	24.39133661
sp|P97447|FHL1_MOUSE	22.5379912	22.21500258
sp|Q7TPM5|SACA9_MOUSE	21.1340896	20.80668884
sp|P19157|GSTP1_MOUSE	29.1555829	28.78454617
sp|P80315|TCPD_MOUSE	24.01402808	23.59265429
sp|P04104|K2C1_MOUSE	28.09666596	27.63797037
sp|P30115|GSTA3_MOUSE	28.51637857	28.02460216
sp|Q3THK7|GUAA_MOUSE	23.82165086	23.31150599
sp|Q3TTY5|K22E_MOUSE	24.60206294	24.06612169
tr|A0A2R8VHP3|A0A2R8VHP3_MOUSE	26.18525703	25.59259778
sp|P80314|TCPB_MOUSE	24.28446688	23.68601681
sp|Q9D8N0|EF1G_MOUSE	27.64672261	27.03494324
sp|P61089|UBE2N_MOUSE	24.12684094	23.50970797
sp|Q9ER72|SYCC_MOUSE	24.38913731	23.72786221

sp|P97315|CSRP1_MOUSE	23.79952201	23.07702679
sp|Q9R0P9|UCHL1_MOUSE	25.32376616	24.58796578
sp|P15626|GSTM2_MOUSE	31.30581729	30.54974037
sp|P11499|HS90B_MOUSE	24.4154254	23.65641185
sp|P54822|PUR8_MOUSE	24.22969953	23.45792447
tr|A8DUK4|A8DUK4_MOUSE	25.8312238	25.0588091
sp|P35700|PRDX1_MOUSE	26.65208608	25.77688438
sp|P42932|TCPQ_MOUSE	23.90969552	23.02157988
sp|P10126|EF1A1_MOUSE	28.25783732	27.34735958
sp|Q60676|PPP5_MOUSE	24.34214203	23.41201871
sp|P20029|BIP_MOUSE	25.40715315	24.42160803
sp|Q8C0M9|ASGL1_MOUSE	26.14087024	25.10333659
sp|Q8JZV7|NAGA_MOUSE	23.67863319	22.56103749
sp|Q9JHU9|INO1_MOUSE	23.86923475	22.75011976
sp|P17156|HSP72_MOUSE	25.90150452	24.7487109
sp|Q8K2B3|SDHA_MOUSE	24.10798175	22.95172373
sp|Q9WUL7|ARL3_MOUSE	24.25981756	23.10349074
sp|P27786|CP17A_MOUSE	24.79344907	23.63100427
tr|A0A0R4J0I1|A0A0R4J0I1_MOUSE	23.84707002	22.66877122
sp|P60867|RS20_MOUSE	22.96822383	21.71874829
sp|Q61496|DDX4_MOUSE	23.88892083	22.63163013
sp|Q3UFF7|LYPL1_MOUSE	24.07882881	22.80454866
sp|O70325|GPX4_MOUSE	27.56223096	26.28042655
sp|P07724|ALBU_MOUSE	26.71580575	25.42439802
sp|Q99MD6|TRXR3_MOUSE	25.99469344	24.70062805
tr|Q8BVP2|Q8BVP2_MOUSE	24.30631745	22.99086741
sp|Q921F2|TADBP_MOUSE	23.46828867	22.06748518
sp|Q9QUR7|PIN1_MOUSE	27.49391326	26.04674037
sp|P02535|K1C10_MOUSE	27.29983907	25.83994853
sp|Q01853|TERA_MOUSE	22.71684706	21.2511925
sp|P54869|HMCS2_MOUSE	25.18734187	23.67275956
tr|F6WHQ7|F6WHQ7_MOUSE	29.76074675	28.10919095
sp|P48774|GSTM5_MOUSE	30.22423168	28.41084367
sp|P48722|HS74L_MOUSE	25.77820279	23.90594845
sp|Q8BGT8|PHIPL_MOUSE	24.17060504	22.29753471
sp|Q8BHG1|NRDC_MOUSE	23.02577845	21.12977882
sp|P50446|K2C6A_MOUSE	26.26813908	24.2929144
sp|P17563|SBP1_MOUSE	26.09891681	23.85835561
sp|P62827|RAN_MOUSE	26.43241002	24.16894919
sp|Q80YT5|SPT20_MOUSE	25.55295015	23.22660773
sp|Q02053|UBA1_MOUSE	23.07394158	20.68271312
sp|Q9D4D4|TKTL2_MOUSE	24.54627953	22.11691994
sp|Q8R1G2|CMBL_MOUSE	25.61132504	23.14588237
sp|Q3U1J4|DDB1_MOUSE	23.51653031	20.93456321

sp|O35490|BHMT1_MOUSE	27.97551156	25.32423805
sp|P60710|ACTB_MOUSE;sp|P63260|ACTG_MOUSE	29.39903551	26.69489152
sp|Q922B2|SYDC_MOUSE	25.0899933	22.34350728
sp|P52480|KPYM_MOUSE	25.12744122	21.81258945
sp|P24472|GSTA4_MOUSE	30.69162515	26.77337103
sp|P01942|HBA_MOUSE	25.24096269	19.4956207
sp|Q99PU5|ACBG1_MOUSE	24.43979306	18.4872853
sp|Q6PAV2|HERC4_MOUSE	18.4872853	0
sp|P47857|PFKAM_MOUSE	19.339929	0
sp|P46425|GSTP2_MOUSE	20.40412659	0
sp|P47934|CACP_MOUSE	20.62008673	0
sp|Q9WU78|PDC6I_MOUSE	20.71822818	0
sp|Q9Z1Q9|SYVC_MOUSE	20.85193673	0
sp|Q8BFR5|EFTU_MOUSE	20.94996132	0
sp|Q99MW1|STK31_MOUSE	21.24140148	0
sp|Q9QWL7|K1C17_MOUSE	21.31591919	0
sp|Q3U0V1|FUBP2_MOUSE	21.37717819	0
sp|Q2NL51|GSK3A_MOUSE	21.44331102	0
sp|P27546|MAP4_MOUSE	21.49715781	0
sp|Q9QYI3|DNJC7_MOUSE	21.57905124	0
sp|Q9EPK6|SIL1_MOUSE	21.64564166	0
sp|Q9WUP7|UCHL5_MOUSE	21.71165798	0
sp|Q8C262|IIGP5_MOUSE	21.78888119	0
sp|Q9WUM5|SUCA_MOUSE	21.86155382	0
sp|Q9WVK4|EHD1_MOUSE	21.91081303	0
sp|Q8VCA8|SCRN2_MOUSE	21.98290788	0
sp|P50516|VATA_MOUSE	22.03266734	0
sp|P32037|GTR3_MOUSE	22.07052851	0
sp|Q8CIG8|ANM5_MOUSE	22.11393543	0
sp|Q8VEK3|HNRPU_MOUSE	22.15032062	0
sp|Q99JY0|ECHB_MOUSE	22.19483118	0
sp|P16381|DDX3L_MOUSE;sp|Q62167|DDX3X_MOUSE	22.27741069	0
sp|Q8R050|ERF3A_MOUSE	22.30501187	0
sp|Q922X9|ANM7_MOUSE	22.3475771	0
sp|P38647|GRP75_MOUSE	22.37985579	0
sp|P60843|IF4A1_MOUSE	22.43491948	0
sp|Q8VDM6|HNRL1_MOUSE	22.48326309	0
sp|Q03265|ATPA_MOUSE	22.57296928	0
sp|Q9Z2K1|K1C16_MOUSE	22.60325966	0
sp|Q8VBT9|ASPC1_MOUSE	22.63709878	0
sp|Q9JLV5|CUL3_MOUSE	22.66836235	0
sp|O35737|HNRH1_MOUSE	22.69580522	0
sp|Q9DA79|DPEP3_MOUSE	22.74000558	0
sp|Q924M7|MPI_MOUSE	22.77809636	0

sp|P55264|ADK_MOUSE	22.81022063	0
sp|P63087|PP1G_MOUSE	22.83466433	0
sp|P16627|HS71L_MOUSE	22.89433022	0
sp|Q68FL4|SAHH3_MOUSE;sp|Q80SW1|SAHH2_MOUSE	22.92336921	0
sp|O55023|IMPA1_MOUSE	22.93999192	0
sp|P80313|TCPH_MOUSE	23.00311392	0
sp|P17710|HXK1_MOUSE	23.0469757	0
sp|Q921F4|HNRLL_MOUSE	23.13309596	0
sp|P10852|4F2_MOUSE	23.15563305	0
sp|P29341|PABP1_MOUSE	23.19055109	0
sp|P07309|TTHY_MOUSE	23.22976634	0
sp|Q8R127|SCPDL_MOUSE	23.27209334	0
sp|Q9R0P3|ESTD_MOUSE	23.30288992	0
sp|Q99KI0|ACON_MOUSE	23.34084333	0
sp|A6H630|ARMT1_MOUSE	23.58820729	0
sp|Q62188|DPYL3_MOUSE	23.66502052	0
sp|P17182|ENOA_MOUSE	23.77132767	0
sp|Q61656|DDX5_MOUSE	23.93928079	0
sp|P24549|AL1A1_MOUSE	24.37260873	0
sp|P08113|ENPL_MOUSE	24.47731382	0
sp|P12382|PFKAL_MOUSE;sp|Q9WUA3|PFKAP_MOUSE	24.62299927	0
sp|Q61316|HSP74_MOUSE	24.64956237	0
sp|Q99KB8|GLO2_MOUSE	24.85605304	0
sp|P61979|HNRPK_MOUSE	24.89832377	0
sp|O88844|IDHC_MOUSE	24.92259074	0
sp|Q64514|TPP2_MOUSE	24.95842588	0
tr|Q9D2T6|Q9D2T6_MOUSE	25.04228548	0
sp|Q9JII5|DAZP1_MOUSE	25.2940143	0
tr|Q9Z1A1|Q9Z1A1_MOUSE	25.48211448	0
sp|Q00612|G6PD1_MOUSE	25.67663102	0
sp|Q6IFX2|K1C42_MOUSE	26.06606779	0
sp|Q61990|PCBP2_MOUSE	27.02208755	0
tr|Q792Z1|Q792Z1_MOUSE	27.40122694	0
