# Supplementary material for: Intracellular domain of CATSPER1 could serve as a cytoplasmic platform for redox processes in mammalian sperm
Source: Anim Biosci. 2024 Dec 13;38(4):655–64. doi: 10.5713/ab.24.0631 (PMC11917441; doi:10.5713/ab.24.0631)
Supplement: Supplementary file 2 [file ab-24-0631-Supplementary-Table-2.pdf]

Supplementary Table S2. Functional annotation of the mCATSPER1-N150 interactome in the testis

Category	No.	Term	Fold Enrichment	pValue	Genes
Biological Process	BP_01	GO:0006749~glutathione metabolic process	45.9803	4.0594E-06	GSTM4, GSTM3, GSTA2, GSTM7, GSTM6
	BP_02	GO:0032212~positive regulation of telomere maintenance via telomerase	59.9058	3.9116E-05	CCT6A, TCP1, MAPK1, CCT5
	BP_03	GO:0061077~chaperone-mediated protein folding	56.6676	4.6314E-05	CCT6A, TCP1, FKBP4, CCT5
	BP_04	GO:0050821~protein stabilization	14.7655	4.9930E-05	CCT6A, HSP90AA1, TCP1, PARK7, GAPDH, CCT5
	BP_05	GO:1904851~positive regulation of establishment of protein localization to telomere	157.2526	1.5378E-04	CCT6A, TCP1, CCT5
	BP_06	GO:0006979~response to oxidative stress	18.0750	1.6066E-04	PRDX2, PRDX5, PRDX4, PARK7, ATOX1
	BP_07	GO:0000226~microtubule cytoskeleton organization	14.9764	3.2934E-04	TUBA1B, TUBB5, TUBB4B, TUBA3A, GAPDH
	BP_08	GO:0006457~protein folding	17.9205	1.3946E-03	CCT6A, HSP90AA1, TCP1, CCT5
	BP_09	GO:0042744~hydrogen peroxide catabolic process	52.4175	1.4512E-03	PRDX2, PRDX5, PRDX4
	BP_10	GO:0045454~cell redox homeostasis	47.6523	1.7551E-03	PRDX2, PRDX5, PRDX4
	BP_11	GO:0051973~positive regulation of telomerase activity	47.6523	1.7551E-03	HSP90AA1, TCP1, MAPK1
	BP_12	GO:0000278~mitotic cell cycle	16.3805	1.8037E-03	TUBA1B, TUBB5, TUBB4B, TUBA3A
	BP_13	GO:0007017~microtubule-based process	44.9293	1.9730E-03	TUBA1B, TUBB5, TUBB4B
	BP_14	GO:0007339~binding of sperm to zona pellucida	33.4580	3.5334E-03	CCT6A, TCP1, CCT5
	BP_15	GO:0006096~glycolytic process	31.4505	3.9897E-03	TPI1, GAPDHS, GAPDH
	BP_16	GO:0006094~gluconeogenesis	30.2409	4.3083E-03	TPI1, GAPDH, FBP1
	BP_17	GO:0042307~positive regulation of protein import into nucleus	29.1209	4.6383E-03	HSP90AA1, MAPK1, IPO5
	BP_18	GO:0034614~cellular response to reactive oxygen species	26.6530	5.5128E-03	PRDX5, MAPK1, PARK7
	BP_19	GO:0018916~nitrobenzene metabolic process	349.4503	5.6125E-03	GSTM4, GSTM7
	BP_20	GO:0030043~actin filament fragmentation	262.0877	7.4765E-03	CFL1, DSTN
	BP_21	GO:0006006~glucose metabolic process	20.4224	9.2268E-03	TPI1, GAPDHS, GAPDH
	BP_22	GO:0070301~cellular response to hydrogen peroxide	20.1606	9.4583E-03	CFL1, AKR1B1, PARK7
	BP_23	GO:0006886~intracellular protein transport	7.7944	1.4083E-02	ARF3, ARF1, ARF2, IPO5
	BP_24	GO:0034599~cellular response to oxidative stress	14.9764	1.6652E-02	PRDX2, PRDX5, PARK7
	BP_25	GO:0042743~hydrogen peroxide metabolic process	116.4834	1.6745E-02	PRDX2, PARK7
	BP_26	GO:0030836~positive regulation of actin filament depolymerization	116.4834	1.6745E-02	CFL1, DSTN
	BP_27	GO:0044598~doxorubicin metabolic process	87.3626	2.2265E-02	AKR1CL, AKR1B1
	BP_28	GO:0006825~copper ion transport	87.3626	2.2265E-02	FKBP4, ATOX1
	BP_29	GO:0044597~daunorubicin metabolic process	87.3626	2.2265E-02	AKR1CL, AKR1B1
	BP_30	GO:0051014~actin filament severing	80.6424	2.4098E-02	CFL1, DSTN
	BP_31	GO:0046166~glyceraldehyde-3-phosphate biosynthetic process	80.6424	2.4098E-02	TPI1, GAPDH
	BP_32	GO:0030042~actin filament depolymerization	80.6424	2.4098E-02	CFL1, DSTN
	BP_33	GO:0002753~cytoplasmic pattern recognition receptor signaling pathway	65.5219	2.9578E-02	YWHAE, IPO5
	BP_34	GO:0061621~canonical glycolysis	61.6677	3.1398E-02	TPI1, GAPDH
	BP_35	GO:0071356~cellular response to tumor necrosis factor	10.5539	3.1917E-02	CFL1, MAPK1, ASS1
	BP_36	GO:0015031~protein transport	4.0824	3.2214E-02	ARF3, ARF1, ARF2, NASP, IPO5
	BP_37	GO:0006878~intracellular copper ion homeostasis	55.1764	3.5027E-02	ARF1, ATOX1
	BP_38	GO:0071475~cellular hyperosmotic salinity response	52.4175	3.6837E-02	AKR1B1, FBP1
	BP_39	GO:0033554~cellular response to stress	52.4175	3.6837E-02	PRDX2, PRDX4
	BP_40	GO:0030521~androgen receptor signaling pathway	47.6523	4.0447E-02	MAPK1, FKBP4
	BP_41	GO:0042178~xenobiotic catabolic process	41.9340	4.5836E-02	GSTM4, GSTM7
	BP_42	GO:0008652~amino acid biosynthetic process	38.8278	4.9413E-02	PHGDH, ASS1
	BP_43	GO:0005975~carbohydrate metabolic process	8.2331	4.9987E-02	LANCL1, PGP, FBP1
Cellular Component	CC_01	GO:0005737~cytoplasm	3.2466	3.6845E-18	ARF3, YWHAE, CSTB, ARF1, ARF2, AKR1CL, AKR1B1, GLRX, PARK7, TUBA3A, IPO5, UBE2L3, LDHC, PRDX2, PRDX5, TUBA1B, PRDX4, TUBB5, PCBP1, CFL1, MAPK1, RPL38, CLIC1, GLUL, AAMDC, CCT5, GSTM4, GSTM3, HSP90AA1, TPI1, LANCL1, TOMM34, FABP9, DSTN, TUBB4B, ASS1, CCT6A, NASP, TCP1, PGP, GAPDHS, FKBP4, GSTM7, GAPDH, FBP1, GSTM6
	CC_02	GO:0005829~cytosol	4.1919	7.9008E-16	YWHAE, CSTB, ARF1, AKR1CL, AKR1B1, PARK7, IPO5, LDHC, PRDX2, PRDX5, TUBA1B, PRDX4, TUBB5, PCBP1, CFL1, MAPK1, RPL38, GLUL, GSTM4, GSTM3, HSP90AA1, TPI1, TOMM34, FABP9, PDIA6, ATOX1, ASS1, CCT6A, SUCLA2, GSTA2, GAPDHS, FKBP4, GSTM7, GAPDH, FBP1, GSTM6
	CC_03	GO:0043209~myelin sheath	30.8567	1.9967E-12	PRDX2, HSP90AA1, TUBA1B, SUCLA2, TCP1, PHGDH, TUBB4B, GAPDH, GLUL, CCT5, ASS1
	CC_04	GO:0005739~mitochondrion	4.6914	2.4413E-07	YWHAE, DUT, HSP90AA1, TOMM34, GLRX, PARK7, ASS1, LDHC, PRDX2, PRDX5, PRDX4, SUCLA2, MAPK1, FKBP4, GAPDH, GLUL, CLIC1
	CC_05	GO:0045171~intercellular bridge	30.7205	1.4238E-06	GSTM4, GSTM3, TUBB5, TUBB4B, GSTM7, GSTM6
	CC_06	GO:0044297~cell body	25.7253	3.4163E-06	CCT6A, TUBB5, TCP1, PARK7, GLUL, CCT5
	CC_07	GO:0005874~microtubule	11.4958	5.2958E-06	CCT6A, TUBA1B, TUBB5, TCP1, FKBP4, TUBB4B, TUBA3A, CCT5
	CC_08	GO:0005832~chaperonin-containing T-complex	158.2105	1.5193E-04	CCT6A, TCP1, CCT5
	CC_09	GO:0015630~microtubule cytoskeleton	12.0404	7.4829E-04	TUBA1B, TUBB5, TUBB4B, TUBA3A, GAPDH
	CC_10	GO:0001669~acrosomal vesicle	12.1234	4.2194E-03	CCT6A, TCP1, FABP9, TCP11
	CC_11	GO:0005856~cytoskeleton	3.3168	4.8051E-03	TUBA1B, TUBB5, CFL1, TCP1, MAPK1, FKBP4, TUBB4B, GAPDH, CCT5
	CC_12	GO:0048471~perinuclear region of cytoplasm	4.3025	5.1278E-03	ARF3, PRDX5, HSP90AA1, AKR1B1, PARK7, FKBP4, CLIC1
	CC_13	GO:0045298~tubulin complex	351.5789	5.5786E-03	TUBB5, TUBB4B
	CC_14	GO:0032991~protein-containing complex	3.9189	7.9840E-03	ARF1, HSP90AA1, TUBB5, NASP, MAPK1, FKBP4, GLUL
	CC_15	GO:0042470~melanosome	15.9809	1.4731E-02	YWHAE, HSP90AA1, PDIA6
	CC_16	GO:0098978~glutamatergic synapse	3.9161	1.7128E-02	YWHAE, ARF1, CFL1, DSTN, UBE3B, GAPDH
	CC_17	GO:0002199~zona pellucida receptor complex	95.8852	2.0306E-02	CCT6A, TCP1
	CC_18	GO:0014069~postsynaptic density	6.5309	2.2410E-02	ARF1, PCBP1, MAPK1, RPL38
	CC_19	GO:0098793~presynapse	6.2043	2.5581E-02	DSTN, RPL38, PARK7, UBE2L3

	CC_20	GO:0072686~mitotic spindle	11.3821	2.7782E-02	TUBB5, MAPK1, TUBB4B
	CC_21	GO:0005634~nucleus	1.5764	3.0302E-02	YWHAE, CSTB, DUT, HSP90AA1, AKR1CL, FABP9, GLRX, PARK7, IPO5, UBE2L3, PRDX5, TUBB5, NASP, PCBP1, CFL1, MAPK1, FKBP4, GAPDH, FBP1, CLIC1
	CC_22	GO:0043025~neuronal cell body	3.9122	3.6787E-02	HSP90AA1, CFL1, GLRX, FKBP4, ASS1
	CC_23	GO:0005929~cilium	4.8944	4.6431E-02	LDHC, GAPDHS, TUBA3A, TCP11
	CC_24	GO:0005790~smooth endoplasmic reticulum	36.3702	5.2663E-02	PRDX4, PDIA6
Molecular Function	MF_01	GO:0043295~glutathione binding	153.7105	3.2737E-10	GSTM4, GSTM3, GSTA2, LANCL1, GSTM7, GSTM6
	MF_02	GO:0004364~glutathione transferase activity	90.4180	5.7569E-09	GSTM4, GSTM3, GSTA2, LANCL1, GSTM7, GSTM6
	MF_03	GO:0005525~GTP binding	11.8239	7.6414E-07	ARF3, ARF1, HSP90AA1, TUBA1B, ARF2, TUBB5, FKBP4, TUBB4B, TUBA3A
	MF_04	GO:0000166~nucleotide binding	4.4374	3.4712E-06	ARF3, ARF1, HSP90AA1, ARF2, TUBB4B, ASS1, UBE2L3, CCT6A, TUBA1B, TUBB5, SUCLA2, TCP1, MAPK1, GLUL, CCT5
	MF_05	GO:0140662~ATP-dependent protein folding chaperone	68.3158	2.6133E-05	CCT6A, HSP90AA1, TCP1, CCT5
	MF_06	GO:0019899~enzyme binding	8.8149	2.9324E-05	GSTM4, YWHAE, GSTM3, KRT79, PARK7, GAPDH, GSTM7, GSTM6
	MF_07	GO:0005515~protein binding	2.3273	3.0345E-05	YWHAE, ARF1, PARK7, IPO5, UBE2L3, LDHC, PRDX2, PRDX5, TUBA1B, PRDX4, PCBP1, CFL1, MAPK1, GLUL, TCP11, CCT5, HSP90AA1, TPI1, TOMM34, ASS1, CCT6A, NASP, TCP1, FKBP4, GAPDH
	MF_08	GO:0140824~thioredoxin-dependent peroxiredoxin activity	307.4211	3.5979E-05	PRDX2, PRDX5, PRDX4
	MF_09	GO:0031625~ubiquitin protein ligase binding	10.4565	4.9740E-05	YWHAE, HSP90AA1, TUBA1B, TPI1, TUBB5, TCP1, UBE2L3
	MF_10	GO:0008379~thioredoxin peroxidase activity	219.5865	7.5369E-05	PRDX2, PRDX5, PRDX4
	MF_11	GO:0051920~peroxiredoxin activity	219.5865	7.5369E-05	PRDX2, PRDX4, PARK7
	MF_12	GO:0042802~identical protein binding	3.4940	1.0673E-04	YWHAE, GSTM4, DUT, HSP90AA1, PARK7, ASS1, PRDX5, PRDX4, MAPK1, GAPDH, FBP1, GLUL, GSTM7, GSTM6
	MF_13	GO:0044183~protein folding chaperone	38.6693	1.4614E-04	CCT6A, HSP90AA1, TCP1, CCT5
	MF_14	GO:0016491~oxidoreductase activity	6.7976	1.4911E-04	LDHC, PRDX2, PRDX5, PRDX4, GAPDHS, AKR1B1, PHGDH, GAPDH
	MF_15	GO:0003924~GTPase activity	9.6069	3.7000E-04	ARF3, ARF1, TUBA1B, ARF2, TUBB5, TUBB4B
	MF_16	GO:0005200~structural constituent of cytoskeleton	26.9668	4.2510E-04	TUBA1B, TUBB5, TUBB4B, TUBA3A
	MF_17	GO:0051082~unfolded protein binding	22.0374	7.6681E-04	CCT6A, HSP90AA1, TCP1, CCT5
	MF_18	GO:0016209~antioxidant activity	61.4842	1.0531E-03	PRDX2, PRDX5, PRDX4
	MF_19	GO:0042803~protein homodimerization activity	4.8739	1.0886E-03	GSTM4, GSTM3, HSP90AA1, TPI1, GSTA2, PARK7, GSTM7, GSTM6
	MF_20	GO:0000287~magnesium ion binding	10.5861	1.2048E-03	ARF1, DUT, SUCLA2, PGP, GLUL
	MF_21	GO:0016740~transferase activity	3.1592	1.9015E-03	GSTM4, GSTM3, GSTA2, LANCL1, MAPK1, UBE3B, GAPDH, GLUL, GSTM7, GSTM6, UBE2L3
	MF_22	GO:0005524~ATP binding	3.3314	2.4553E-03	CCT6A, HSP90AA1, SUCLA2, TCP1, MAPK1, FKBP4, GLUL, CCT5, ASS1, UBE2L3
	MF_23	GO:0004601~peroxidase activity	35.7466	3.1005E-03	PRDX2, PRDX5, PRDX4
	MF_24	GO:0005504~fatty acid binding	30.7421	4.1701E-03	GSTA2, FABP9, GSTM7
	MF_25	GO:0051287~NAD binding	27.9474	5.0244E-03	GAPDHS, PHGDH, GAPDH
	MF_26	GO:0032767~copper-dependent protein binding	341.5789	5.7416E-03	FKBP4, ATOX1
	MF_27	GO:0097110~scaffold protein binding	16.8913	1.3259E-02	YWHAE, HSP90AA1, PARK7
	MF_28	GO:0004365~glyceraldehyde-3-phosphate dehydrogenase (NAD+) (phosphorylating) activity	128.0921	1.5239E-02	GAPDHS, GAPDH
	MF_29	GO:1903136~cuprous ion binding	113.8596	1.7128E-02	PARK7, ATOX1
	MF_30	GO:0003824~catalytic activity	6.8316	1.9931E-02	LDHC, SUCLA2, GLUL, FBP1
	MF_31	GO:0019903~protein phosphatase binding	13.0263	2.1628E-02	YWHAE, HSP90AA1, CFL1
	MF_32	GO:0016874~ligase activity	11.1384	2.8907E-02	SUCLA2, GLUL, ASS1
	MF_33	GO:0016853~isomerase activity	10.2474	3.3681E-02	TPI1, FKBP4, PDIA6
	MF_34	GO:0001758~retinal dehydrogenase activity	56.9298	3.3968E-02	AKR1CL, AKR1B1
	MF_35	GO:0016787~hydrolase activity	2.5178	3.5260E-02	ARF1, DUT, HSP90AA1, TUBA1B, PGP, PARK7, TUBA3A, FBP1
	MF_36	GO:0004032~aldose reductase (NADPH) activity	48.7970	3.9518E-02	AKR1CL, AKR1B1
	MF_37	GO:0048156~tau protein binding	44.5538	4.3200E-02	HSP90AA1, FKBP4
	MF_38	GO:0016620~oxidoreductase activity, acting on the aldehyde or oxo group of donors, NAD or NADP as acceptor	40.9895	4.6869E-02	GAPDHS, GAPDH
	MF_39	GO:0016887~ATP hydrolysis activity	4.8337	4.7868E-02	CCT6A, HSP90AA1, TCP1, CCT5
